# Immunohistochemical insights into Saffold virus infection of the brain of juvenile AG129 mice

**DOI:** 10.1186/s12985-016-0654-8

**Published:** 2016-11-25

**Authors:** Shawn Zheng Kai Tan, Mookkan Prabakaran

**Affiliations:** Temasek Life Science Laboratory, 1 Research Link, National University of Singapore, Singapore, 117604 Republic of Singapore

**Keywords:** Saffold virus, Apoptosis, Demyelination, Juvenile brain

## Abstract

**Background:**

Saffold Virus (SAFV) is a human cardiovirus that is suspected of causing infection of the central nervous system (CNS) in children. While recent animal studies have started to elucidate the pathogenesis of SAFV, very little is known about the mechanisms behind it.

**Method:**

In this study, we attempted to elucidate some of the mechanisms of the pathogenesis of SAFV in the brain of a juvenile mouse model by using immunohistochemical methods.

**Results:**

We first showed that SAFV is able to infect both neuronal and glial cells in the brain of 2 week-old AG129 mice. We then showed that SAFV is able to induce apoptosis in both neuronal and glial cells in the brain. Lastly, we showed that SAFV infection does not show any signs of gross demyelination in the brain.

**Conclusion:**

Overall, our results provide important insights into the mechanisms of SAFV in the brain.

**Electronic supplementary material:**

The online version of this article (doi:10.1186/s12985-016-0654-8) contains supplementary material, which is available to authorized users.

## Findings

Saffold Virus (SAFV) is a novel human cardiovirus identified in 2007 from the stool sample of a child with a fever of unknown origin [1]. Phylogenetic analysis revealed that SAFV is closely related to Theiler’s murine encephalomyelitis virus (TMEV) [2, 3], a virus well studied for its neurovirulence [4]. Since then, SAFV have been isolated in children from multiple continents [1, 5–11] and have been shown to be highly prevalent in humans [7]. Recently, SAFV was found in the cerebrospinal fluid (CSF) of a previously healthy child that experienced sudden death [12], and in stool samples of children with non-polio acute flaccid paralysis [6], suggesting that the virus might be able to cause serious invasive infection of the central nervous system (CNS) of children [11]. TMEV, a virus similar to SAFV, is divided into two strains based on their neurovirulence after intracerebral inoculation [4]; the GDVII strain causes acute fatal poliomyelitis, killing all infected mice within 2 weeks, while DA strain causes milder encephalomyelitis, which then progresses to persistent infection and progressive demyelination reminiscent of multiple sclerosis [13]. It has been suggested that infection and apoptosis of neurons are responsible for fatal outcomes, while persistence in and subsequent apoptosis of glial cells such as oligodendrocytes is responsible for persistent demyelination [14], suggesting that the cell type that the virus infects and persist in hugely determines the outcome of infection.

In this short study, we wanted to examine the infectivity of SAFV in the juvenile (2 week old) brain by studying the cell types it infects. We further attempted to elucidate the effects of the infection. In order to do that, we used AG129 mice which we have previously shown to be a good model for SAFV infection in the CNS [15]. We used previously isolated genotype 3 SAFV [16] as a model of SAFV infection due to its high prevalence [7]. Infectious clone used in this study was obtained as previously described in Tan et al. [15]. We then studied brain tissue of infected animals using immunohistochemical methods to elucidate some of the mechanisms involved in the pathogenesis of SAFV. Brain tissue used in this study were either from AG129 mice (B and K Universal, North Humberside, UK), or FVB/NTac mice (Haplotype H2^q^) (*InVivos* Pte Ltd. Singapore). All animal experimental protocols were approved by the Institutional Animal Care and Use Committee (IACUC) of the Temasek Life Sciences Laboratory, Singapore (IACUC approval number TLL-14-025). Two week-old AG129 mice (n = 6) and 3–4 week old AG129 mice (n = 6) were inoculated i.p. with 5 × 10^5^ TCID_50_/mouse of SAFV. Mice were sacrificed on day 6 or 35 (days post infection) dpi as described below. FVB/NTac mice (n = 6) were inoculated intracranial (I.C.) with 5 × 10^5^ TCID_50_/mouse of SAFV. To obtain brains, AG129 mice were anesthetized intraperitoneal (i.p.) with Ketamine (100 mg/kg)/Xylazine (20 mg/kg) and transcardially perfused with 50 ml phosphate-buffered saline (PBS) followed by 100 ml 4% paraformaldehyde (PFA) in PBS. The mice were then decapitated and the brains and spinal cord were removed and post-fixed in 4% PFA overnight at 4 °C. Brains were then transferred to a solution of 20% sucrose in PBS and stored overnight at 4 °C before freezing over liquid nitrogen and stored at −80 °C. The brains were then embedded in Shandon M-1 Embedding Matrix (Thermo, Kalamazoo, MI, USA) and sectioned through a cryostat (16 um).

**Fig. 1:**
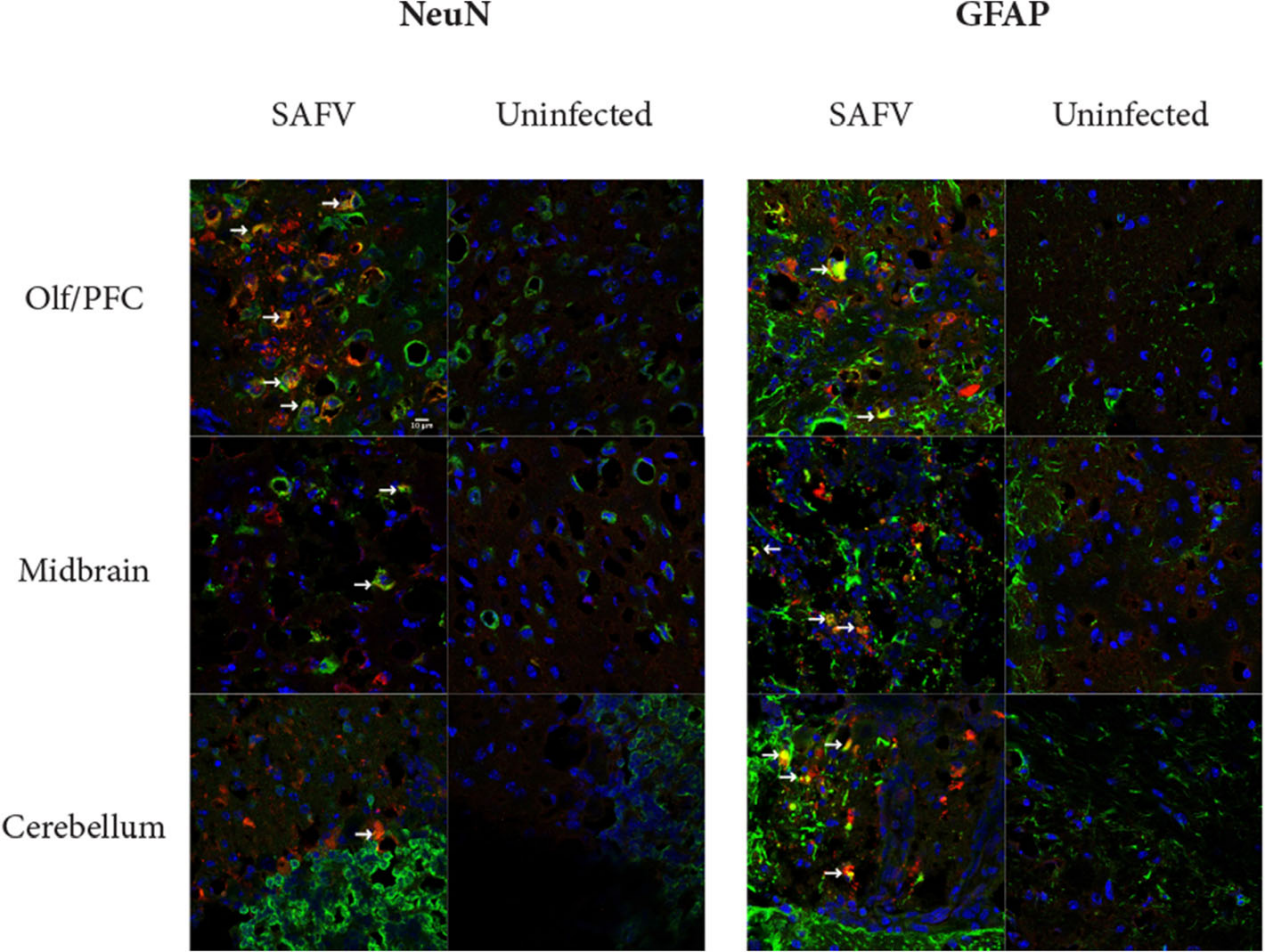
Immunohistochemical co-localisation of SAFV VP1 with NeuN or GFAP. Brain sections of 6dpi SAFV infected 2 week-old AG129 mice were stain with anti-SAFV VP1 and anti-NeuN or anti-GFAP. Stained SAFV infected or uninfected mice brain sections were viewed and taken with a confocal fluorescence microscope. SAFV VP1 is labelled in red, NeuN or GFAP is labelled in green, and DAPI is labelled in blue. Arrows point at examples of colocalisation

To assess the cell type that SAFV infects in the brain, double labelling immunohistochemistry was carried out on brains of 2 week-old AG129 mice inoculated i.p with SAFV, and sacrificed 6 dpi. Briefly, tissue sections were incubated with rabbit anti-SAFV VP1 [15] and guinea pig anti-glial fibrillary acidic protein (GFAP) (Synaptic Systems, Goettingen, Germany) or guinea pig anti-neuronal nuclei (NeuN) (Synaptic Systems) overnight at 4 °C. Tissue sections were then further incubated with secondary antibody Alexa Fluor 594 conjugated anti-rabbit IgG and Alexa Fluor 488 conjugated anti-guinea pig IgG (Invitrogen, Gaithersburg, USA) and counterstained with DAPI (Invitrogen, Gaithersburg, USA) before coverslipping with anti-fade mounting medium (DAKO, Copenhagen, Denmark). Resulting stains were viewed under a Leica TCS-SPE II confocal microscope. Double labelling immunohistochemistry of infected brains revealed that SAFV VP1 signal was found in the olfactory/prefrontal region (Olf/PFC), midbrain, and cerebellum, and was colocalised in both GFAP and NeuN positive cells (Fig. 1 & Additional file 1: Figure S1), indicating that the virus infects both neuronal and glial cells, but neither exclusively. Our results agree with Sorgeloos et al. [16] who showed infection of both neuronal and glial cells, but not with Kotani et al. [17] who showed infection of glial cells, but not neuronal cells, in both early adult (6 week-old) and neonatal brains of ddY and BALB/c mice. However it should be noted that Sorgeloos et al. [16] showed a preference to glial cells over neuronal cells. We believe that our results are not contradictory, but rather compliments the data shown in Kotani et al. [16] by highlighting the varied outcomes seen in different strains of virus, mice breed, and age.

**Fig. 2:**
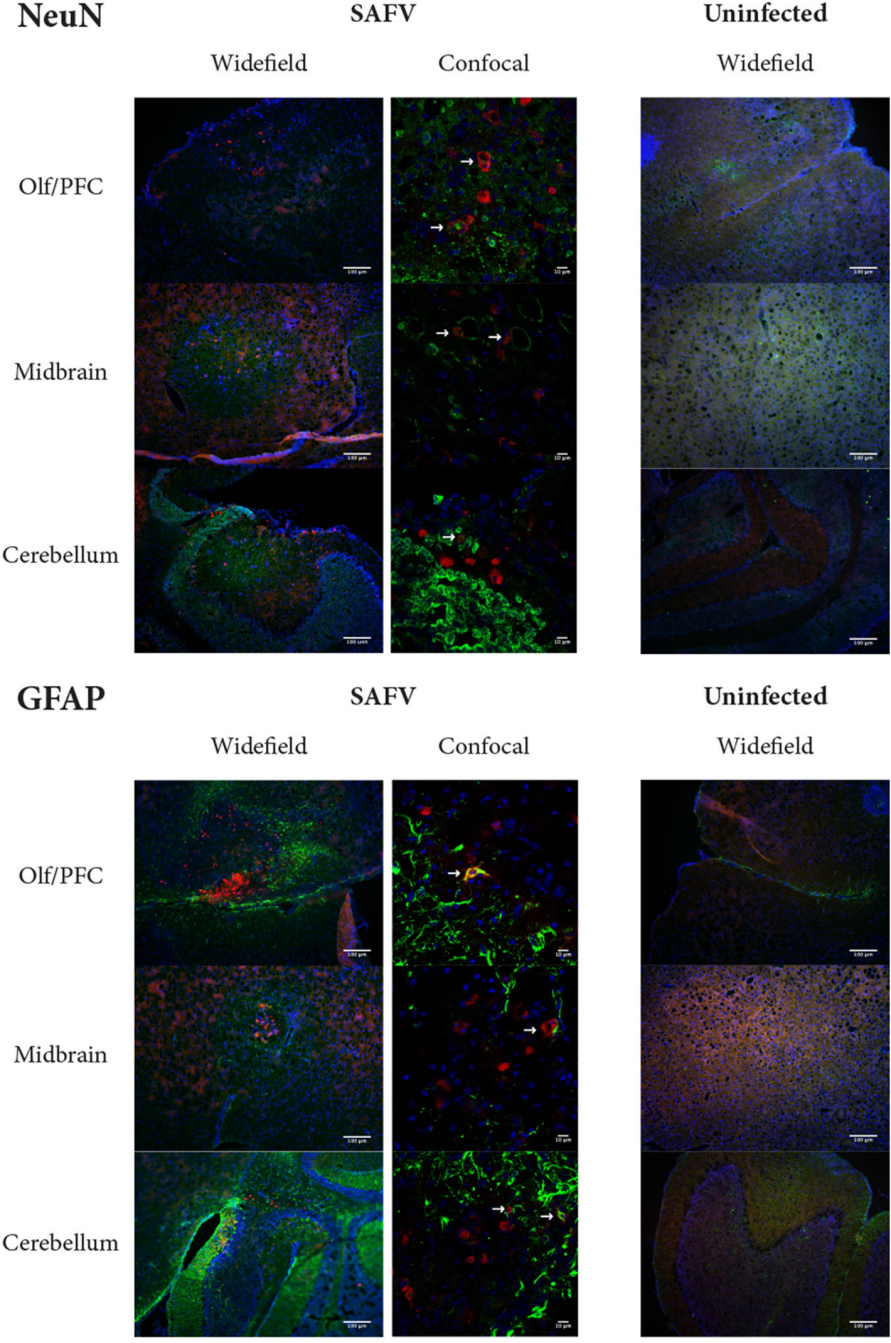
Immunohistochemical co-localisation of cleaved caspase 3 with NeuN or GFAP. Brain sections tissue of 6 dpi SAFV infected or uninfected 2 week-old AG129 mice were stained with anti-cleaved caspase 3 and anti-GFAP or anti-NeuN. Resulting stain was viewed and taken with widefield or confocal fluorescence microscope. Cleaved caspase 3 is labelled in red, NeuN or GFAP is labelled in green, and DAPI is labelled in blue. Arrows point at examples of colocalisation

In order to test if SAFV causes apoptosis in both neuronal and glial cells, we used cleaved (activated) caspase 3 as a marker for apoptosis (due to its involvement in both intrinsic and extrinsic pathways), detecting it through immunohistochemistry, and co-staining it with either NeuN or GFAP (Fig. 2) antibodies as markers for neuronal and glial cells respectively. The same immunohistochemistry protocol as above was used, except primary antibodies used were anti-cleaved caspase 3 (Synaptic Systems) and guinea pig anti-GFAP or guinea pig anti-NeuN (Synaptic Systems). Resulting stains were viewed under upright fluorescence microscopy (Zeiss, Jena, Germany) and a Leica TCS-SPE II confocal microscope. SAFV infected brains showed apoptosis in similar regions as viral infections, and colocalisation with both GFAP and NeuN positive cells were observed. Control uninfected brains showed no cleaved caspase 3 staining (Fig. 2). This was unsurprising as we did expect the virus to behave similarly (in that they induced apoptosis) to previous cell culture work [18], it was however an important finding in understanding how the virus was affecting the brain.

**Fig. 3:**
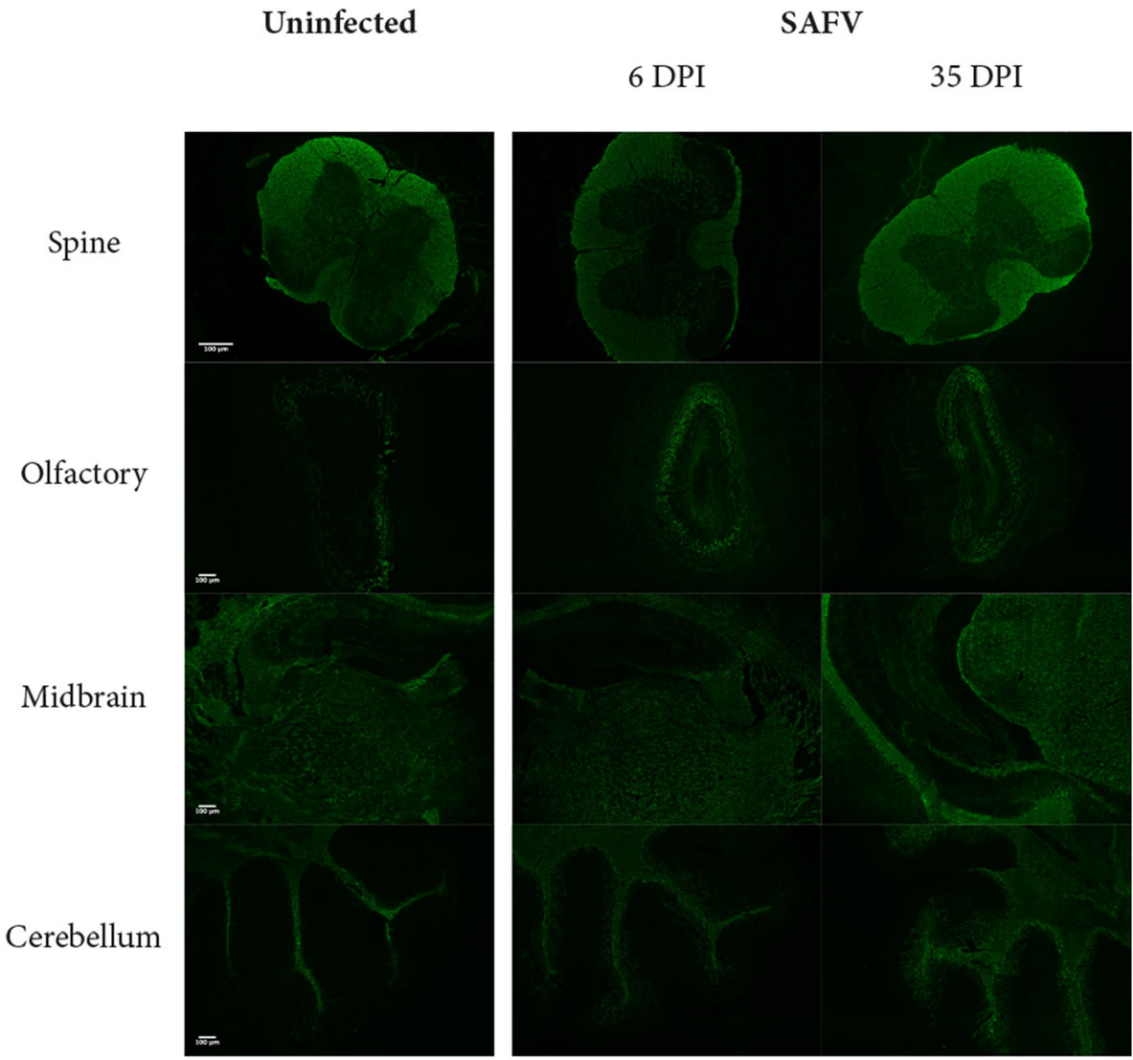
Fluorescence images of MBP stained uninfected and SAFV infected brain. Brain sections of 6dpi 2 week-old AG129 mice or 35dpi 3–4 week-old AG129 mice were stained with anti-MBP antibodies. No observable demyelination was detected in any sections

Given that TMEV is able to cause multiple sclerosis like demyelination [19], and that SAFV does infect and induce apoptosis in glial cells, we wanted to see if SAFV was able to cause multiple sclerosis like demyelination. Myelin Basic Protein (MBP) was used as a marker for myelin and was detected through immunohistochemistry, a method that has been shown to be highly sensitive for detection of myelin [20], using the same protocol as above. We found no signs of gross demyelination in 2 week-old AG129 mice on 6 dpi (Fig. 3). In order to see if SAFV infection can cause demyelination subsequently, we infected 3–4 week-old AG129 mice with SAFV, an age which we have previously shown that mice initially only show minor symptoms, but subsequently are able to survive infection, showing no further observable symptoms [15]. Mice were then sacrificed at 35 dpi and brain tissue underwent immunohistochemistry using anti-MBP (Abcam, Cambridge, UK), which similarly revealed no signs of gross demyelination (Fig. 3). In order to ensure that the lack of demyelination was not due to the use of an inappropriate model, FVB/NTac mice (Haplotype H2^q^) were inoculated I.C. with SAFV, similar to previously established demyelination model with TMEV. Mice showed no symptoms to 50 dpi. Brains similarly showed no signs of demyelination (data not shown). Our results were counter intuitive given that we showed that SAFV is able to both infect, and induce apoptosis, in glial cells. We would hence expect oligodendrocytes to be affected, causing demyelination reminiscent of multiple sclerosis. It should however be noted that we did not stain specifically for oligodendrocytes, but rather took a more general approach of staining for a general glial marker GFAP. Kotani et al. [16], who similarly showed SAFV infection of GFAP positive cells, however showed no viral infection of oligodendroglial suggesting viral induced apoptosis of oligodendrocytes is unlikely to happen in the brain of SAFV infected animal. This could explain why we did not observe any demyelination.

Information about SAFV infection has increased in the past few years, with data concentrated on respiratory and gastrointestinal tract infections [11], however data informing CNS infection remains relatively low, despite its importance shown by clinical samples of SAFV found in children with neurological symptoms [6, 11, 12]. In this short paper, we examined SAFV infection of the brain in an animal model and presented important new insights to the neuropathogenesis of SAFV. We showed that SAFV is able to infect both neuronal and glial cells in the developing brain of a mouse model, causing apoptosis in both. We however showed no gross demyelination in the brains of the infected animals. We hence provided important insights, extending the findings of our previous paper [15], and provided a strong basis on which the mechanisms of neuropathogenesis of SAFV can be further examined.

## Additional file

Additional file 1: Figure S1. High magnification micrograph of Immunohistochemical stain showing co-localisation of SAFV VP1 with NeuN or GFAP. Brain sections tissue of 6dpi SAFV infected 2 week-old AG129 mice were stain with anti-SAFV VP1 and anti-NeuN or anti-GFAP. Stained SAFV infected or uninfected mice brain sections were viewed and taken with a confocal fluorescence microscope. SAFV VP1 is labelled in red, NeuN or GFAP is labelled in green, and DAPI is labelled in blue. (JPG 297 kb)
