# Supplementary figures and images for: Immunohistochemical insights into Saffold virus infection of the brain of juvenile AG129 mice
Source: Virol J. 2016 Nov 25;13:191. doi: 10.1186/s12985-016-0654-8 (PMC5123230; doi:10.1186/s12985-016-0654-8)

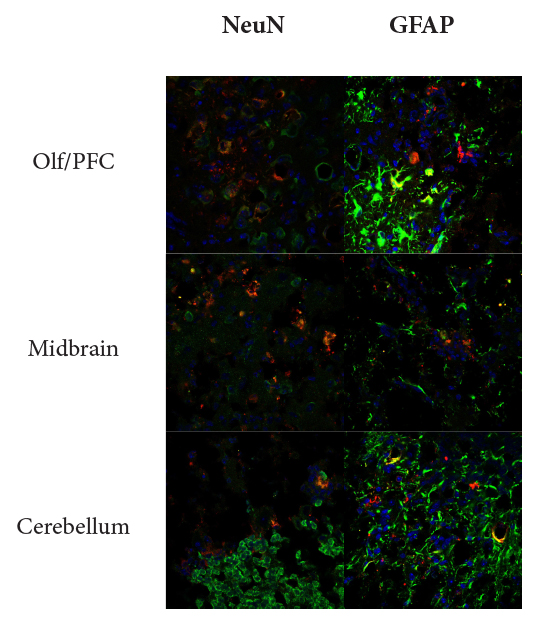

Supplement: Additional file 1: Figure S1. — High magnification micrograph of Immunohistochemical stain showing co-localisation of SAFV VP1 with NeuN or GFAP. Brain sections tissue of 6dpi SAFV infected 2 week-old AG129 mice were stain with anti-SAFV VP1 and anti-NeuN or anti-GFAP. Stained SAFV infected or uninfected mice brain sections were viewed and taken with a confocal fluorescence microscope. SAFV VP1 is labelled in red, NeuN or GFAP is labelled in green, and DAPI is labelled in blue. (JPG 297 kb) [file 12985_2016_654_MOESM1_ESM.jpg]
